# Supplementary material for: Biohybrid lung Development: Towards Complete Endothelialization of an Assembled Extracorporeal Membrane Oxygenator
Source: Bioengineering (Basel). 2023 Jan 5;10(1):72. doi: 10.3390/bioengineering10010072 (PMC9854558; doi:10.3390/bioengineering10010072)
Supplement: Supplementary file 1 [file bioengineering-10-00072-s001.zip › bioengineering-2065876-supplementary.docx]

**
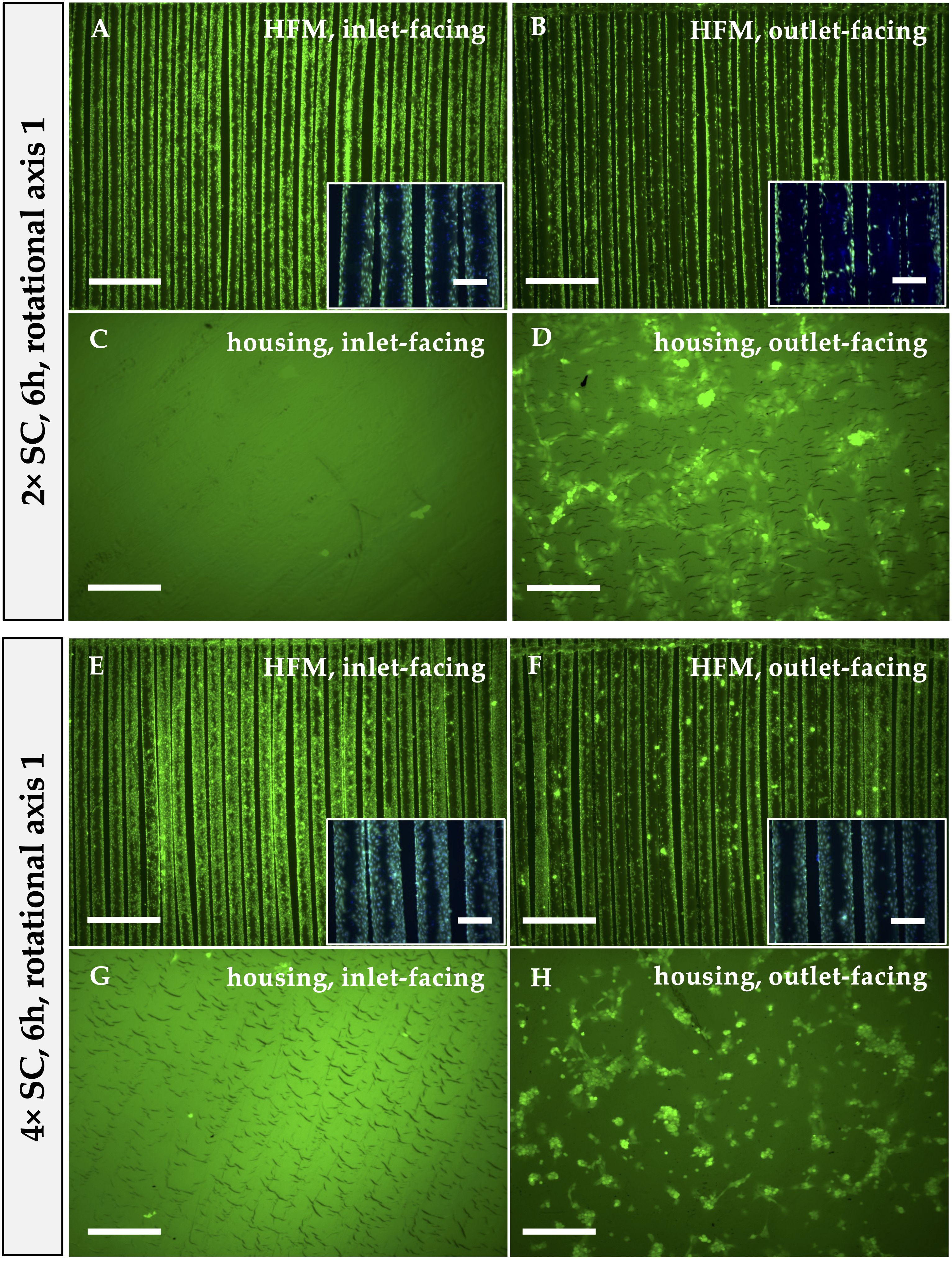
**

**Figure S1.** Improved seeding efficiency depended on applied EC concentration. Fluorescence microscopy was performed on HFMs (**A**,**B**,**E**,**F**) and the housing (**C**,**D**,**G**,**H**), facing the inlet (**A**,**C**,**E**,**G**) and the outlet (**B**,**D**,**F**,**H**), after seeding with 2 × SC (**A**–**D**) or 4 × SC (**E**–**H**) and using calcein (green)/Hoechst 33342 (blue) staining. Scale: 2 mm; insert boxes in A, B, E, F, scale: 400 µm.
